# Systems Biology: New Clue to Heat Stress

**Published:** 2005-02

**Authors:** Carol Potera

The human body cools itself by increasing blood flow to surface areas and by sweating. When these cooling responses fail, the result may be heat-related injuries such as heat stroke or even death. Although the consequences of impaired heat loss are known, the complex networks that increase skin blood flow during heat stress are not well understood. Now, however, a new study identifies one type of histamine receptor as a key regulator of body temperature.

Some people are particularly prone to heat illnesses, including the elderly and those with diabetes, heart disease, and cystic fibrosis. No one knows if global warming will bring more catastrophic heat waves, but the number of at-risk people will increase as the population ages. In order to protect at-risk individuals during heat waves, researchers must identify all the steps involved in skin blood flow. “Right now, we don’t fully understand all the interactions that control skin blood flow during heat stress in healthy young people,” says study coauthor Brett Wong, a doctoral student in the Department of Human Physiology at the University of Oregon in Eugene.

Wong and his colleagues put 11 healthy volunteers in specially designed suits fitted with tubing that circulates hot water and quickly warms the body. Once the participants’ core body temperatures reached about 100°F, they were dermally dosed with either the antihistamine pyrilamine, which acts through the H1 receptor; the antihistamine cimetidine, which acts through the H2 receptor; or nitro-L-arginine-methyl ester (L-NAME), which blocks nitric oxide. It’s well documented that nitric oxide raises skin blood flow when the body heats up, and that L-NAME blocks blood flow. All three chemicals act locally in the skin and do not circulate in the body.

The team used noninvasive laser-Doppler flowmetry to measure how these agents affected skin blood flow during heat stress. As expected, L-NAME blocked skin blood flow. Cimetidine’s blockage of the H2 receptor showed no effect. But when pyrilamine blocked the H1 receptor, blood flow to the skin was dramatically reduced. The report appears in the November 2004 issue of the *Journal of Physiology*.

Although pyrilamine is an over-the-counter antihistamine, it’s premature to say that people taking antihistamines for allergies may have trouble regulating their body temperature in heat stress, Wong cautions. Future studies will look at oral antihistamines, heat stress, and skin blood flow.

Studies like Wong’s “are slowly putting the pieces of the puzzle together,” says Michael J. Joyner, vice chair of the Department of Physiology at the Mayo Clinic. Once the basic biology is laid out, researchers can test what drugs help or hinder skin blood flow. In the future, “we may be able to warn at-risk people to either avoid or take certain drugs during heat waves,” says Joyner. Outdoor workers or athletes who compete in hot climates will also benefit from such advice.

